# Inhibiting PRMT5 induces DNA damage and increases anti-proliferative activity of Niraparib, a PARP inhibitor, in models of breast and ovarian cancer

**DOI:** 10.1186/s12885-023-11260-z

**Published:** 2023-08-18

**Authors:** Shane O’Brien, Michael Butticello, Christine Thompson, Boris Wilson, Anastasia Wyce, Vivek Mahajan, Ryan Kruger, Helai Mohammad, Andy Fedoriw

**Affiliations:** 1 Tumor Cell Targeting RU, GlaxoSmithKline, Collegeville, USA; 2 Synthetic Lethality RU, GlaxoSmithKline, Collegeville, USA; 3 GlaxoSmithKline, 1250 South Collegeville Road, Collegeville, PA 19426 USA

**Keywords:** Combinations, Breast, Ovarian, PARP, Protein methyltransferase 5

## Abstract

**Background:**

Inhibitors of **P**oly (**A**DP-**R**ibose) **P**olymerases (PARP) provide clinical benefit to patients with breast and ovarian cancers, by compromising the DNA repair activity of cancer cells. Although these agents extend progression-free survival in many patients, responses can be short lived with many patients ultimately progressing. Identification of combination partners that increase dependence of cancer cells to the DNA repair activity of PARPs may represent a strategy to increase the utility of PARP inhibitors. Protein arginine methyltransferase 5 (PRMT5) regulates DNA damage response pathways through splicing and protein modification, and inhibitors of PRMT5 have recently entered clinical trials.

**Methods:**

The effect of PRMT5 inhibition on the levels of DNA damage and repair markers including γH2AX, RAD51, and 53BP1 was determined using high content immunofluorescent imaging. The anti-proliferative activity of the combination of PRMT5 and PARP inhibitors was evaluated using in vitro models of breast and ovarian cancers using both cell lines and ex vivo patient derived xenografts. Finally, the combinations of PRMT5 and PARP inhibitors were evaluated in cell line xenograft models in vivo.

**Results:**

Inhibition of PRMT5 by GSK3326595 led to increased levels of markers of DNA damage. The addition of GSK3326595 to the PARP inhibitor, niraparib, resulted in increased growth inhibition of breast and ovarian cancer cell lines and patient derived spheroids. In vivo, the combination improved the partial effects on tumor growth inhibition achieved by either single agent, producing complete tumor stasis and regression.

**Conclusion:**

These data demonstrate that inhibition of PRMT5 induced signatures of DNA damage in models of breast and ovarian cancer. Furthermore, combination with the PARP inhibitor, Niraparib, resulted in increased anti-tumor activity in vitro and in vivo. Overall, these data suggest inhibition of PRMT5 as a mechanism to broaden and enhance the clinical application of PARP inhibitors.

**Supplementary Information:**

The online version contains supplementary material available at 10.1186/s12885-023-11260-z.

## Background

**P**oly (**A**DP-**R**ibose) **P**olymerases (PARP) are involved in a range of biological processes including stress response, chromatin remodeling, and the DNA damage response, specifically at sites of single-stranded DNA breaks [ref. 1]. Upon inhibition of PARP activity, these lesions cannot be repaired, leading to the formation of double stranded DNA breaks which can be repaired through either the accurate homologous recombination (HR) pathway, or by error prone non-homologous end joining (NHEJ) [ref. 2]. In cells with a deficient HR pathway, repair of DSBs defaults to NHEJ, where continued accumulation of inaccurately repaired lesions ultimately results in a loss of viability [ref. 3]. As cancer therapies, PARP inhibitors have demonstrated clinical benefit for patients with mutations in genes that are essential to HR, including *BRCA1* or *BRCA2* [ref. 4]. In addition to preventing the repair of single stranded breaks, PARP inhibitors trap PARP enzymes on chromatin, producing additional DNA damage and further accelerating genomic instability [refs. 5, 6, 7]. Despite the clinical success of PARP inhibitors, many patients fail to respond resulting in a short-lived therapeutic response [refs. 8, 9]. Therefore, combinations are being explored to increase the duration and quality of responses of PARP inhibitors to additional patient populations, including those with a proficient HR pathway [refs. 10, 11–13].

**PR**otein Arginine **M**ethyl**T**ransferases (PRMTs) have diverse roles in cellular biology through methylation of arginine residues on a large number of proteins. Arginine methylation exists in three forms, mono-methylated (MMA), asymmetric dimethylated (ADMA), and symmetric dimethylated (SDMA), and each state of methylation can impact protein function or localization [refs. 14, 15]. The majority of cellular SDMA is catalyzed by PRMT5, and [ref. 16]. overexpression of PRMT5 is associated with poor prognosis and survival in glioblastoma, breast, ovarian, and gastric cancers (refs. 17, 18–20). Consequently, several inhibitors of PRMT5 have been developed, including GSK3326595 and JNJ-64619178, and are currently under investigation in clinical trials [refs. 20, 21, 22].

PRMT5 mediated methylation regulates spliceosome assembly and function, transcriptional silencing through histone methylation, and tumor suppressor activity [refs. 23, 24, 25]. PRMT5 contributes to the regulation of the DNA damage response, by methylation of DNA damage repair proteins or alternative splicing of their transcripts. Arginine methylation by PRMT5 is required for the stability of a 53BP1, a protein required for NHEJ, thereby attenuating its activity [ref. 26]. In hematopoietic cells, PRMT5 regulates the splicing of the histone acetyltransferase TIP60 to promote utilization of the HR pathway, and the shift in TIP60 splicing isoforms incurred by PRMT5 loss increased the usage of NHEJ [refs. 27, 28]. PRMT5 modulation also impairs homologous recombination by depleting RPA, leading to a vulnerability to DNA damage by gemcitabine [ref. 29]. Therefore, modulating DNA damage response and repair proteins through PRMT5 inhibition may increase the sensitivity of cancer cells to PARP inhibition. To explore this hypothesis, we assessed the effect of the a potent and selective, clinical PRMT5 inhibitor, GSK3326595, on DNA damage response pathways and evaluated efficacy in combination with the PARP inhibitor, niraparib, in HR-proficient human cancer models. In vitro, PRMT5 inhibition increased markers of DNA damage in breast and ovarian cancer cell lines, and combination with PARP inhibitor produced additive to synergistic growth inhibition in both cancer cell lines and 3D clonogenic patient derived xenograft (PDX) models. In vivo, combination treatment resulted in tumor stasis and regression in xenograft models of breast and ovarian cancer, respectively, whereas each single agent only partially affected tumor growth. Together, this work demonstrates the utility of targeting PRMT5 activity to expand the therapeutic benefit of PARP inhibition.

## Methods

### Cell lines and compounds

Cell lines were obtained from various repositories and licensed accordingly. NIH cell lines were obtained and supplied by the Department of Health and Human Services and Drs. Gazdar and Minna. All cell lines were maintained in the recommended cell culture media at 37 °C and in 5% CO_2_. Identity of all cell lines was validated by short tandem repeat profiling, and each cell line was confirmed negative for mycoplasma using the ATCC universal Mycoplasma Detection Kit. GSK3326595 and niraparib were obtained from GSK medicinal chemistry and prepared at stocks of 40 mM in 100% DMSO. Etoposide (Millipore, MA) was prepared as a 20 mM stock in 100% DMSO.

### Immunofluorescence and high content imaging

Cells were seeded 24 h prior to compound treatment in triplicate, in clear bottom 96-well plates (Perkin Elmer, MA). Cells were treated with either DMSO or a 9 point 3-fold dilution series of GSK3326595 (10,000 nM – 1.52 nM). On the 5th day of compound treatment, cells were treated with either DMSO or 10 µM etoposide. After 24 h, cells were fixed with 4% formaldehyde, washed, and blocked with IF buffer (1X PBS + 0.1% w/v BSA + 0.2% Triton-X, 0.05% Tween-20 + 10% goat serum) for 2 h at room temperature. Cells were then incubated with antibodies against RAD51 (1:1500, Catalog # ab133534, Abcam Cambridge, UK), 53BP1 (1:100, Catalog # 4937, Cell Signalling Technologies, MA), γH2AX (1:500, Catalog # 05-636, Millipore, MA), or anti-geminin (1:250 abcam, Catalog # ab195047, Cambridge, UK or 1:100 Catalog # MABS121, Millipore, MA) and incubated at 4 °C overnight. Cells were washed 4x with IF buffer and stained with anti-mouse alexa 488 (1:1000, Catalog # A32723, Invitrogen, MA), anti-rabbit alexa 633 (1:1000, Catalog # A21071, Invitrogen, MA), and Hoechst (1:2000, ThermoScientific, MA) for 2 h at room temperature, protected from light. Immunofluorescent staining was imaged using the Perkin Elmer Opera Phenix confocal microscope. 39–60 fields of view (FOV) per well of each channel, DAPI, alexa 488, alexa 633 were taken using a 40X water objective. Images were analysed using the Perkin Elmer Harmony software. First, full intact nuclei were identified using the signal from the Hoechst stain. The mean fluorescent intensity (MFI) of nuclear geminin staining was determined where an MFI > 750 was considered geminin positive and < 750 was considered geminin negative. A sliding parabola was used to detect RAD51, 53BP1, and γH2AX spots and a threshold was applied to identify foci. Full nuclei with ≥ 5 RAD51 or 53BP1 foci was considered positive while ≥ 15 γH2AX foci or a γH2AX MFI > 2000 was considered positive. The percent of double positive (marker positive and geminin positive) of the total geminin positive cells per well was calculated. If the total population of geminin negative or geminin positive was fewer than 100 cells, then those populations were excluded from further analysis.

### siRNA mediated gene specific knockdowns

OVCAR3 cells were seeded in 96-well plates at 5 × 10^4^ cells/well. Twenty-four hours after seeding, cells were transfected with either 1 pmol non-targeting siRNA (Dharamacon, Lafayette, CO), BRCA1-specific siRNA (Dharamacon, Lafayette, CO), or TP53BP1-specific siRNA (Dharamacon, Lafayette, CO) using the Lipofectamine RNAi Max (Invitrogen, Waltham, MA), according to the manufacturer’s protocols. Seventy-two hours after transfection, cells were treated with either 0.1% DMSO or 10 µM etoposide for 24 h.

### In vitro growth double titration and long-term proliferation

Double titration proliferation experiments were completed as previously described [ref. 30]. Cell lines were treated with a 2-fold dilution of GSK3326595 or niraparib with concentrations ranging from 10,000 nM to 0.3 nM. Co-treatments involved a dilution of GSK3326595 dosed horizontally across the plate and a dilution of niraparib dosed vertically down the plate. The resulting matrix of drug concentrations allowed for each concentration of GSK3326595 to be plated with each concentration of niraparib. Plates were incubated at 37 °C in 5% CO2 for 10 days. On the tenth day of treatment, cells were lysed with CellTiter-Glo (CTG) (Promega, WI) according to the manufacturer’s protocol and the chemiluminescent signal was detected using the Synergy Neo plate reader (Biotek, VT). Long-term proliferation experiments were completed by seeding cells at low densities in 24-well plates. Cells were then treated with a 5-fold dilution series of GSK3326595 with concentrations ranging from 5000 nM to 8nM alone or in combination with niraparib at 400 nM, 1000 nM, or 2000 nM. 14-days after treatment, cell culture media was removed, and cells were stained with 0.5% w/v crystal violet in 1X PBS with 2.7% paraformaldehyde and 1% methanol for 30 min at room temperature. Stain was removed and plates were washed with continuous flowing water until excess crystal violet removed. Plates were allowed to air dry overnight prior to imaging. Proliferation was measured after 14 days of treatment using CTG as previously described. After treatment, inhibition of cell growth was expressed as a percentage of the number of cells present at the time of compound addition (T_0_) as previously described [refs. 30, 31]. Relative inhibition versus vehicle and the Growth/Death index (GDI), a composite representation cell growth and cell death, were calculated as previously described [30]. Growth death index dose responses were fit using the four parameter curve equation Y = Bottom +(Top-Bottom)/1(1+(IC50/X)^HillSlope^. The Bliss model calculation was employed by determining the expected inhibition of each combination concentration based on the inhibition obtained with the single agent concentrations according to the calculation E*a* + E*b*-Ea*E*b* where E is the effect (inhibition), *a* is GSK3326595 and *b* is niraparib [ref. 32]. Additionally, the highest over single agent model calculation was employed by subtracting the observed relative growth inhibition by the most potent growth effect of either single agent. The difference between the expected and observed from both models were determined where values ≥ 10 were considered to be a synergistic effect, values between − 10 and 10 were additive effect, and values ≥-10 were considered antagonistic [30].

### *Ex-vivo* 3D tumor clonogenic PDX assay

The 3D tumor clonogenic PDX assays were conducted at Charles River Laboratories. 3D PDX spheroids were established from single cell suspensions of PDX tumors plated in 96-well ultra-low attachment plates in 0.4% (w/v) agar (Bd Biosciences, MA) in IMDM, supplemented with 20% fetal calf serum (Sigma-Aldrich, MA) and 50ug/mL gentamicin (Life Technologies, CA). 24 h after plating the soft-agar layer was covered with culture media containing a titration of GSK3326595, with concentrations ranging from 10 µM to 3.16 µM, alone or in combination with fixed concentrations of niraparib at 1.8 µM or 0.57 µM for 8 to 13 days. After the treatment period, viable colonies were stained with 2-(4-iodophenyl)-3-(4-nitrophenyl)-5-phenyltetraoliu chloride (INT) (Sigma-Aldrich, MA) and counted using automated imaging. The number of colonies greater than 50 μm remaining in the treatment group was expressed as a fraction of those in the control arm. Single agent activity of GSK3326595 was determined using a 4-parameter non-linear curve fit. IC_50_ and absolute IC_50_ values were determined from the halfway point from the top or bottom plateau and/or where growth was 50% of control, respectively. The geometric mean of all IC_50_ values was used to determine the potency of GSK3326595. The excess over bliss or HSA was determined from percent of control as previously described.

### In vivo mouse models and tumor growth

MDA-MB-468 xenografts were carried out by GlaxoSmithKline (GSK, PA) and OVCAR3 xenograft models were carried out by Charles River Laboratories (CRL, MA). All mice were maintained in a specific pathogen-free barrier facility at GSK or CRL. All studies were conducted in accordance with the GSK Policy on the Care, Welfare and Treatment of Laboratory Animals and were reviewed by the Institutional Animal Care and Use Committee either at GSK or by the ethical review process at the institution where the work was performed. A single cell suspension of 3 × 10^6^ MDA-MB-468 cells in 100% Matrigel was delivered subcutaneously in the rear flank of female NSG mice. OVCAR-3 xenografts were established by implanting ~ 1mm^3^ tumor fragments of serial subcutaneous engraftments in the rear flank of 10 week old female CD.17 SCID mice (Fox Chase SCID®, CB17/Icr-Prkdcscid/IcrIcoCrl, Charles River, MA). Once tumor growth was evident, tumor volume and body weights were measured twice weekly. Tumor volumes were measured using calipers and tumor volumes were determined using the following formula: tumor volume = (Length x Width^2^)/2. When the mean tumor size reached ~ 150-250mm^3^ for MDA-MB-468 or 100–150 mm^3^ for OVCAR3, animals were randomized, using stratified block randomization, into study groups (n = 10/group) and animals were dosed twice daily with 50 mg/kg GSK3326595, or once daily with 35 mg/kg niraparib, or in combination. Animals were monitored daily, and any clinical observations were recorded. Investigators were not blinded to mouse xenograft studies. An ordinary AVOVA test with a tukey’s multiple comparison test post hoc test was used to determine significance between treatment groups.

## Results

### Inhibition of PRMT5 by GSK3326595 results in the accumulation of markers of DNA damage

**Fig. 1:**
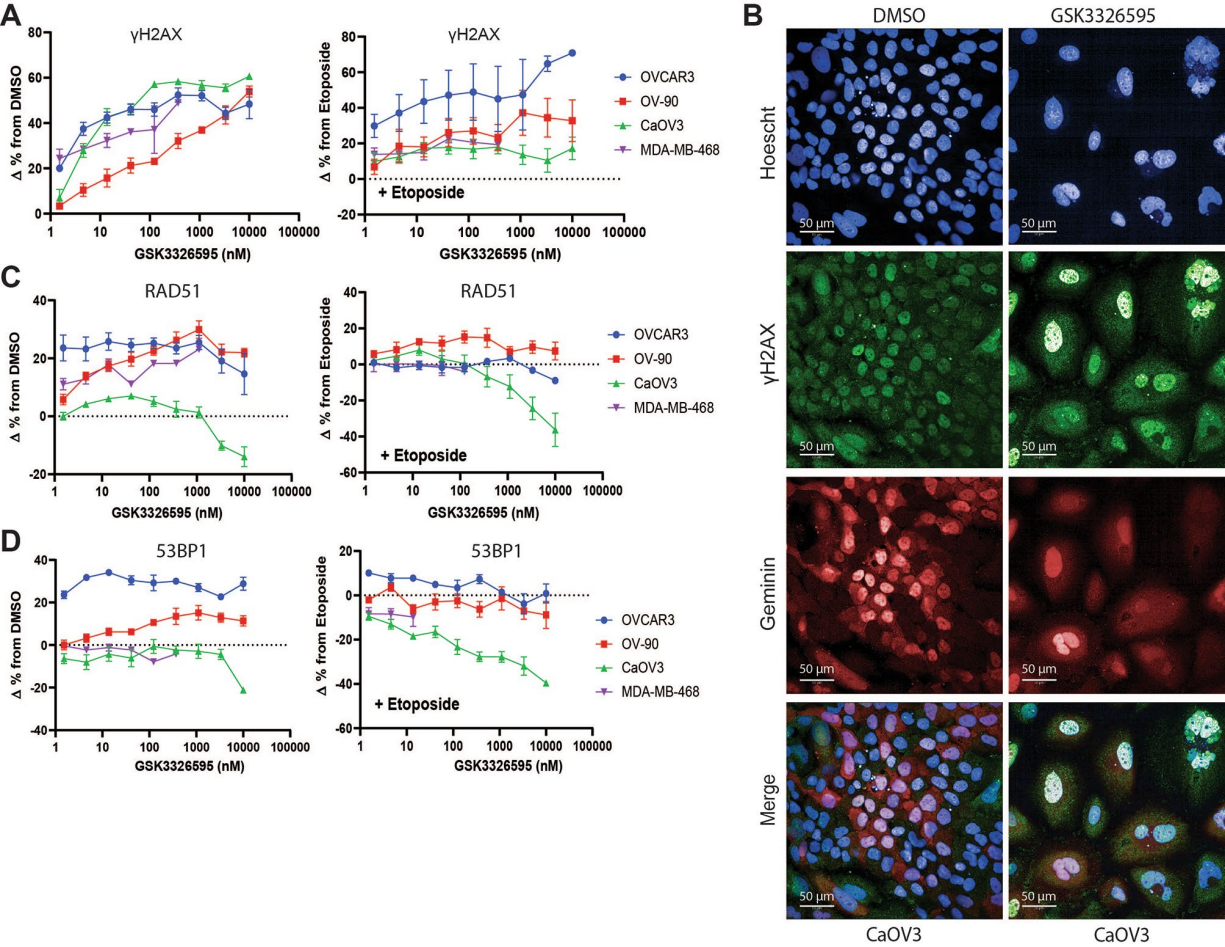
Inhibition of PRMT5 increased markers of DNA damage in replicating cancer cell lines. **A**, The change of the percentage of GSK3326595 treated cells positive for geminin, a marker of replication, and γH2AX, a marker of DNA damage, from DMSO or etoposide treated cells measured by immunofluorescence. Cells with a geminin MFI > 750 were considered geminin positive and cells with an MFI > 2000 or > 15 foci per nuclei were considered γH2AX positive. The change in the percentage of cells positive for both geminin and γH2AX was plotted (n = 2 per cell line with 2 technical replicates per cell line). **B**, Representative immunofluorescent images of CaOV3 cells treated with DMSO or GSK3326595 for 6 days. Cells stained with anti-γH2AX, anti-geminin, and nuclear stain Hoechst. Immunofluorescent images taken using a 40X objective. Scale bar represents 50 μm. **C**, The change in the percentage of GSK3326595-treated cells positive for geminin and RAD51, a marker of HR mediated DNA repair, from DMSO or etoposide-treated cells measured by immunofluorescence. Cells with > 5 foci per nuclei were considered RAD51 positive (n = 2 biological replicates with 2 technical replicates per cell line). **D**, The change in the percentage of GSK3326595 treated cells positive for geminin and 53BP1, a marker of NHEJ mediated DNA repair, from DMSO or etoposide treated cells measured by immunofluorescence. Cells with > 5 foci per nuclei were considered 53BP1 positive (n = 2 per cell line). All error bars represent mean ± SEM.

To evaluate the effect of PRMT5 inhibition on DNA damage, an imaging assay was used to assess levels of DNA damage (phosphorylated histone H2AX, γH2AX) in three ovarian and one breast cancer cell line treated with a dose response of the clinical PRMT5 inhibitor, GSK3326595 (referred to as PRMT5i). In addition to PRMT5i alone, the topoisomerase II inhibitor, etoposide, was added during the final day of treatment to increase DNA damage, providing further stimulus for the activation of DNA damage response and repair pathways. Etoposide treatment alone increased γH2AX positive cells after 24 h of treatment (Supplemental Fig. 1A, D). Interestingly, inhibition of PRMT5 resulted in a dose-dependent accumulation of γH2AX positive cells in all cell lines, and further increased the number of γH2AX positive cells upon etoposide treatment in OVCAR-3 (Fig. 1A, B). Since increased γH2AX and DNA damage was observed, we determined whether DNA repair mechanisms were affected by PRMT5 inhibition. We validated the ability of the high content imaging assay to measure activity of the homologous recombination and NHEJ pathways by quantifying cells with RAD51 and 53BP1 foci after siRNA mediated knockdown of proteins responsible for controlling the choice between homologous recombination, BRCA1, and non-homologous recombination, 53BP1. Since homologous recombination can only be active when a homologous DNA template is available, cells were counterstained for geminin to mark the S/G2 phases of the cell cycle [ref. 33]. OVCAR3 cells were transfected with non-targeting control siRNA, *BRCA1* siRNA, or *TP53BP1* siRNA for 72 h, then treated with the DNA damaging agent, etoposide (Supplemental Fig. 2A). Knockdown of *BRCA1* resulted in the reduction of cells positive for RAD51 with an accompanying increase in 53BP1 positive cells (Supplemental Fig. 1B, C). Knockdown of *TP53BP1* mRNA had the opposite effect resulting in increasing the cells positive for RAD51 while decreasing percentage of 53BP1 positive (Supplemental Fig. 1B, C). The switch reveals the ability of this high content assay to discern the biological consequence of modulation HR mediated DNA repair (Supplemental Fig. 1D, E). 53BP1 and RAD51 foci were monitored after PRMT5 inhibition alone or in combination with etoposide. Consistent with increased DNA damage, etoposide treatment alone increased the frequency of both RAD51 and 53BP1 positive cells in all cell lines tested (Supplemental Fig. 1B-D). Despite increased γH2AX in all cell lines tested, inhibition of PRMT5 alone or in combination with etoposide had variable effects on markers of HR or NHEJ. In CaOV3, an ovarian cancer line, PRMT5 inhibition decreased markers of both HR and NHEJ, despite an increase in H2AX. (Fig. 1C, D). Overall, these data suggest inhibition of PRMT5 results in increased DNA damage, without consistent effects on the markers of the major DNA repair pathways.

### Increased anti-proliferative effects for the combination of GSK3326595 and PARP inhibition compared to single agent treatment

**Fig. 2:**
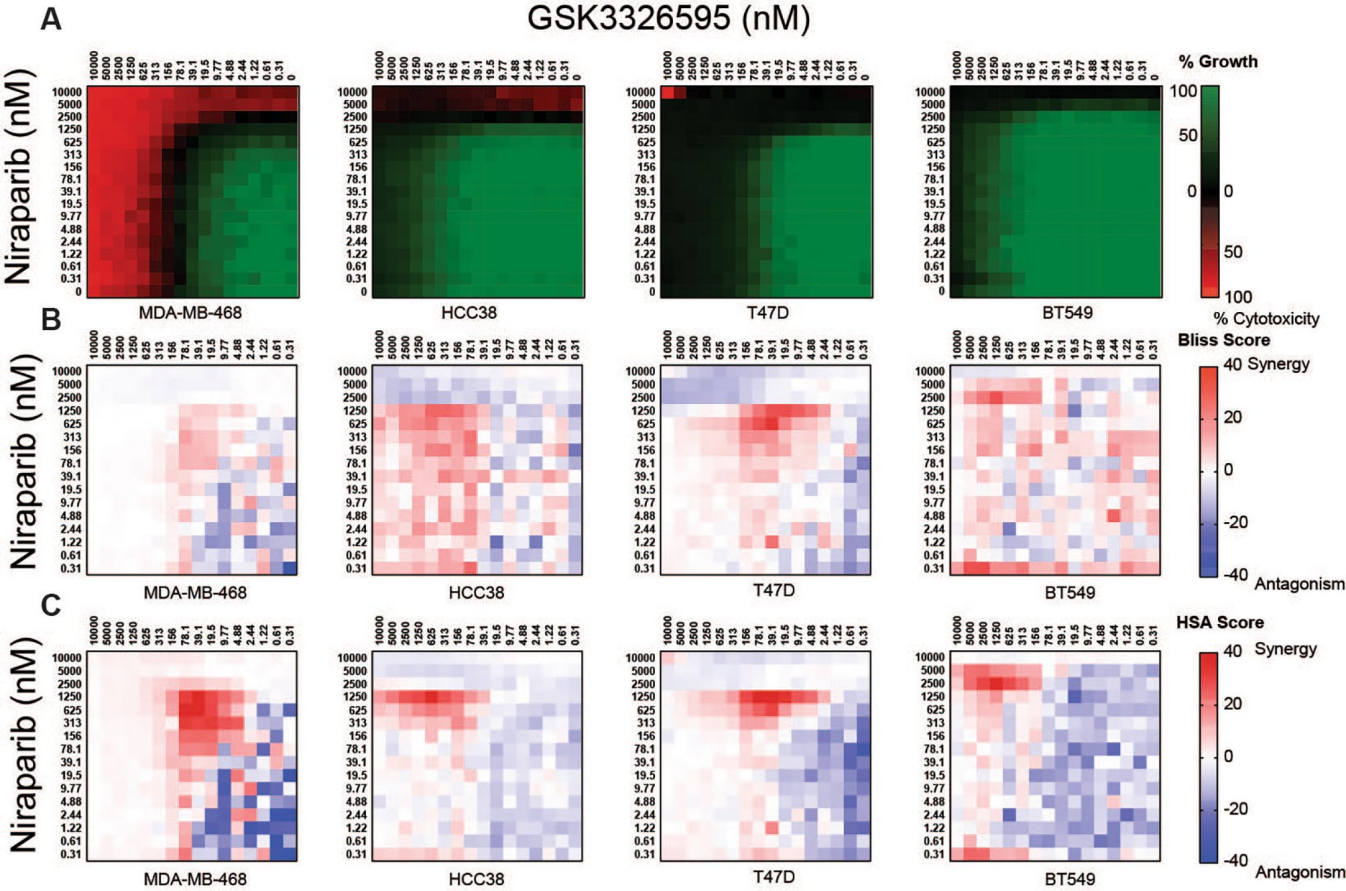
Enhanced anti-proliferative effects following combined PRMT5 and PARP inhibition in breast cancer cell lines. **A**, Average growth death index of breast cancer cell lines treated with the combination of a double titration of GSK3326595 and niraparib. Cell lines were treated with the combination for 10 days and cell growth was determined by CTG (n = 3 per cell line). **B**, Average excess over Bliss synergy scores calculated from growth inhibition of breast cancer cell lines treated with a double titration of GSK3326595 and niraparib. Values ≥ 10 are considered synergy and values ≤-10 considered antagonism. (n = 3 per cell line) **C**, Average excess of HSA synergy scores of breast cancer cell lines treated with a double titration of GSK3326595 and niraparib. Values ≥ 10 are considered synergy and values ≤-10 considered antagonism. (n = 3 per cell line)

**Fig. 3:**
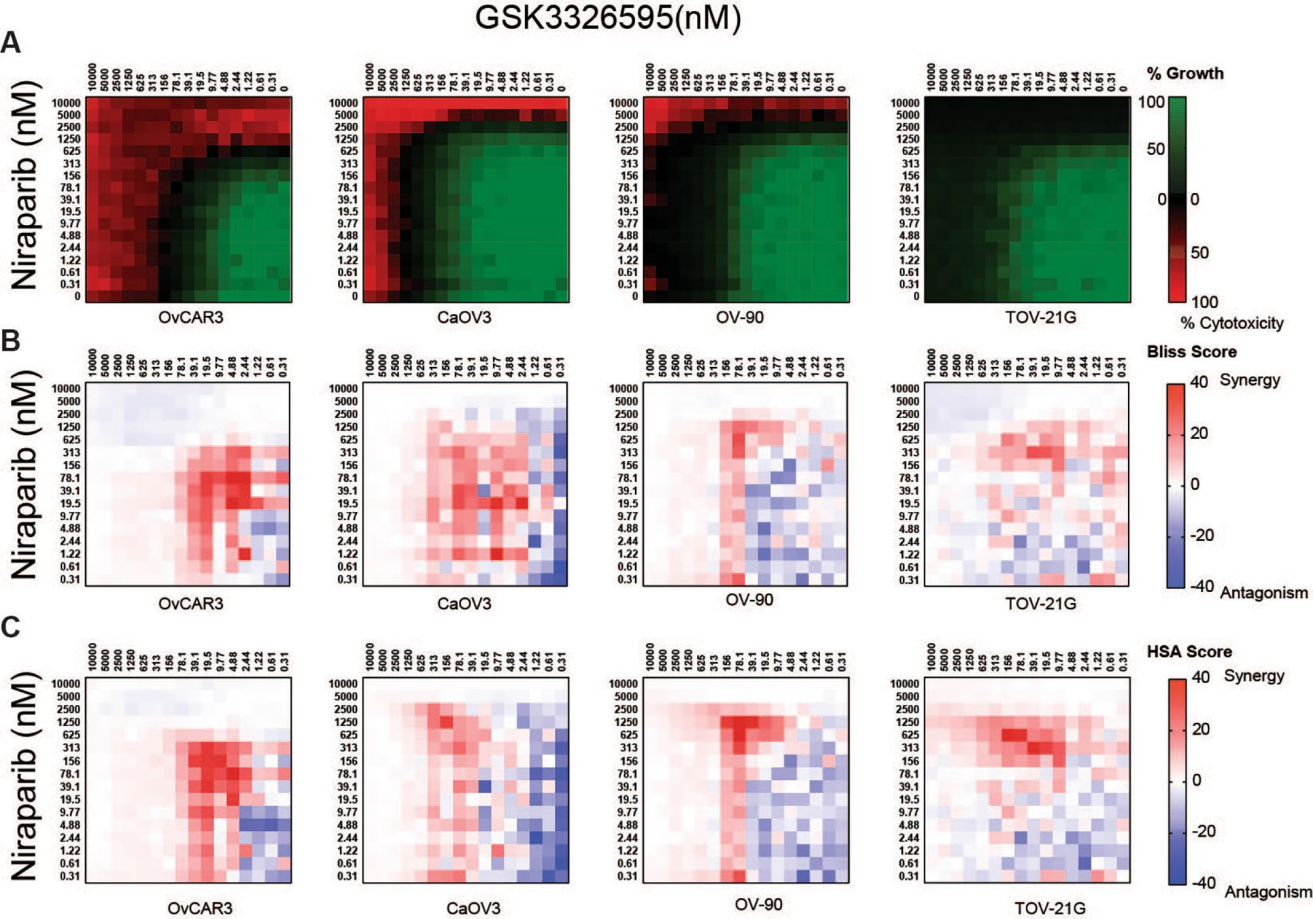
Enhanced anti-proliferative effects following combined PRMT5 and PARP inhibition in ovarian cancer cell lines. **A**, Average growth death index of ovarian cancer cell lines treated with a double titration of GSK3326595 and niraparib. Cell lines were treated with the combination for 10 days and cell growth was determined by CTG (n = 3 per cell line). **B**, Average excess over Bliss synergy scores calculated from growth inhibition of ovarian cancer cell lines treated with a double titration of GSK3326595 and niraparib. **C**, Average excess of HSA synergy scores of ovarian cancer cell lines treated with double titration of GSK3326595 and niraparib. For **B** and **C**, values ≥ 10 considered synergistic and values ≤-10 considered antagonism. (n = 3 per cell line)

Niraparib is a potent inhibitor of PARP activity with the ability to trap PARP enzymes on chromatin, leading to DNA damage and cell death [refs. 34, 35–38]. Niraparib is currently approved for HR-deficient advanced ovarian cancers or as a maintenance therapy for advanced and recurrent platinum sensitive ovarian cancer regardless of HR status [ref. 39]. To determine whether the anti-proliferative activity of niraparib could be enhanced by PRMT5 inhibition, we evaluated the anti-proliferative effects of the combination on homologous recombination proficient breast and ovarian cancer cell lines. We used a double-titration approach to test each concentration of PRMT5i with each concentration of niraparib [refs. 30, 31]. Cell growth was expressed as a percentage of the number of cells present at the time of adding compound and the growth death index (GDI), a composite representation of growth inhibition and cell death relative to the vehicle, was used to evaluate the effect of the combination. Niraparib demonstrated limited activity across cell lines tested, with the gIC_50_ 10 to 100-fold greater than and the sensitive *BRCA1* mutant cell line MDA-MB-436, suggesting these lines can be considered resistant to PARP inhibition (Supplemental Fig. 3A, C). GSK3326595 was a more potent single agent than Niraparib across all cell lines tested (Supplemental Fig. 3B, D). Expression or mutational status of PRMT*5*, *BRCA1*, *BRAC2*, *PARP1*, *TP53BP1*, or *RAD51* did not correlate with sensitivity to either single agent (Supplemental Table 2). However, a cell line described to have loss of HR and high levels of NHEJ, OVCAR3, was the most sensitive cell line to both niraparib and GSK3326595 as single agents, suggesting that the combination could have a beneficial effect on both HR proficient and deficient cancers [ref. 40]. To determine if the effects of the combination on cell growth were synergistic, the Bliss independence (BLISS) or Highest over Single Agent (HSA) models were employed using the relative growth inhibition to determine additive and synergistic effects [refs. 32, 41, 42]. The BLISS independence model was calculated using the equation E*a* + E*b*-Ea*E*b* where E is the effect (inhibition), *a* is GSK3326595 and *b* is niraparib [ref. 32]. The HSA model was calculated by subtracting the observed relative growth inhibition by the most potent growth effect of either single agent. Synergy scores greater than 10 were considered synergistic, scores between − 10 and 10 were additive, and scores less than − 10 were considered antagonistic [refs. 13, 30]. Over 10 days of culture, the combination increased cytotoxicity in 1 of 4 breast cancer cell lines at concentrations where each single agent produced a cytostatic response (Fig. 2A). Synergistic effects on growth inhibition were observed in all breast cancer cell lines in a concentration area ranging between 5000 nM and 9.77 nM GSK3326595 and 5000 nM to 78.1 nM niraparib, using either method of synergy calculation (Fig. 2B, C). Antagonistic effects in the BLISS and HSA models were observed at the lowest or highest concentrations tested where neither compound had an effect or where complete growth inhibition was achieved by single agent treatment, which may reflect small variation around extremes of growth inhibition (Fig. 2B, C). In ovarian cancer cell lines, cytotoxic responses to the combination were observed in three of four cell lines (Fig. 3A). Similar to breast cancer cell lines, the combination produced distinct areas of synergy by either synergy calculation (Fig. 3B, C). The matrix approach demonstrated a combination benefit of PRMT5i and niraparib in both breast and ovarian cancer cell lines, however the majority of cytotoxic responses were observed at high concentrations of either inhibitor.

**Fig. 4:**
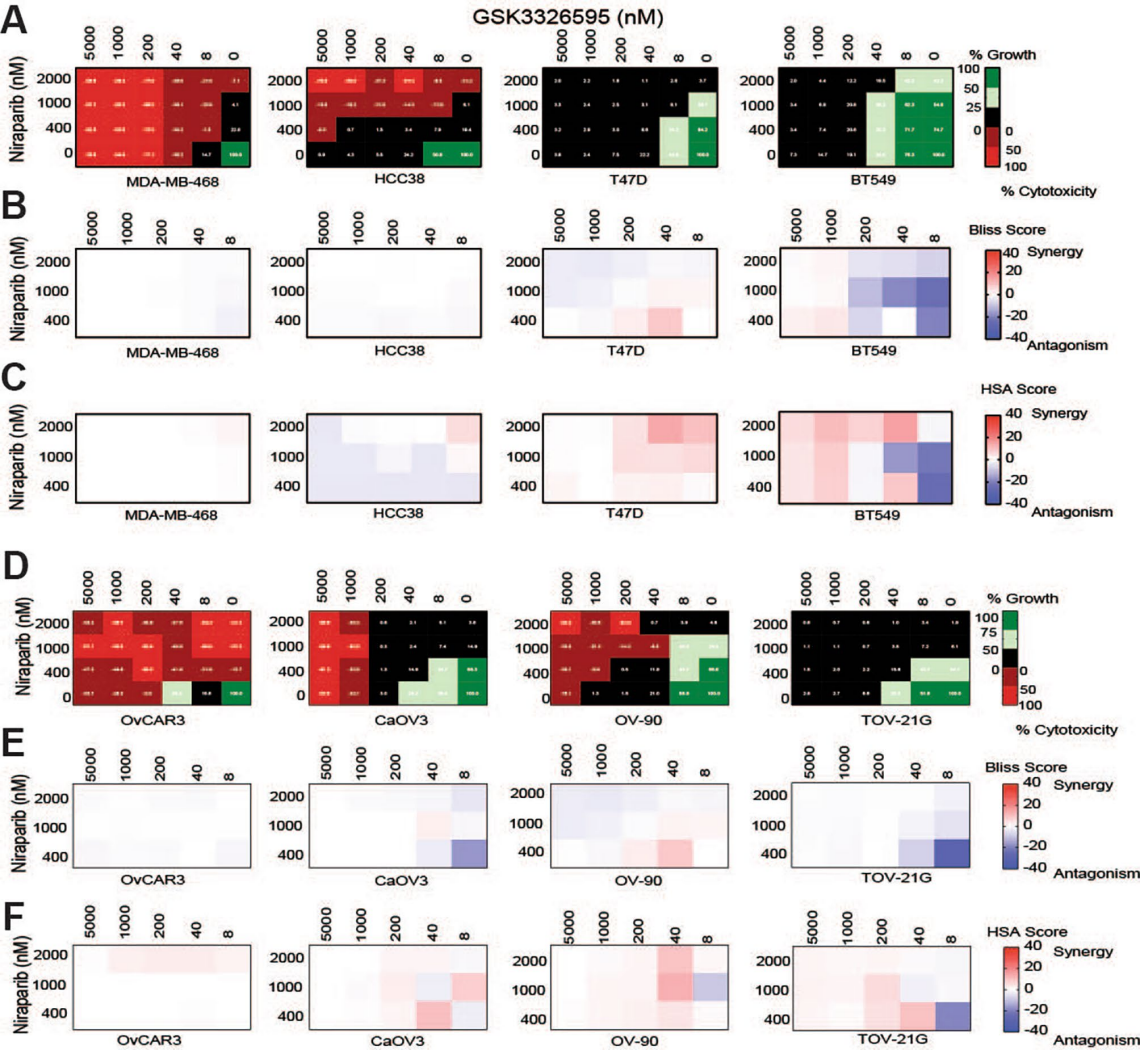
Increased potency on growth inhibition and cytotoxicity by inhibition of PRMT5, PARP, and the combination in breast and ovarian cancer cell lines following long-term exposure. **A**, Average growth death index of breast cell lines treated with a titration of GSK3326595 combined with fixed concentrations of niraparib. Cell lines were seeded at low densities and treated with the combination for 14 days. Cell growth was determined by CTG(n = 2 per cell line). **B**, Average excess over Bliss synergy scores of breast cancer cell lines treated with titration of GSK3326595 and niraparib. Values ≥ 10 considered synergy and values ≤-10 considered antagonism. (n = 2 per cell line) **C**, Average HSA synergy scores of breast cancer cell lines treated with double titration of GSK3326595 and niraparib. Values ≥ 10 considered synergy and values ≤-10 considered antagonism. (n = 2 per cell line). **D**, Average growth death index of ovarian cancer lines treated with the combination of GSK3326595 and niraparib, as described in (A) **E**, Average excess over Bliss synergy scores of ovarian cancer cell lines treated with GSK3326595 and niraparib, as described in (B) **F**, Average excess of HSA synergy scores of ovarian cancer cell lines treated with GSK3326595 and niraparib, as described in C

We hypothesized that increased exposure times to the combination could reveal additional synergistic growth inhibition or cytotoxicity at lower concentrations. To test this, we evaluated a longer duration colony formation assay (CFA) format. The minimal cell to cell contact and low growth signals generates a stressful environment which can be used to assess the in vitro cytotoxic effects of therapies [refs. 43, 44, 45]. We modified the CFA by using Cell Titer Glo (CTG) to more robustly quantify cell growth. Cell lines assessed in the double-titration matrix were seeded at low densities to encourage colony growth then treated GSK3326595, niraparib, or the combination for 14 days. This assay format revealed increased growth inhibition or cytotoxicity for each single agent compared to the 10-day double titration proliferation assay in breast cancer cell lines (Fig. 4A, Supplemental Fig. 4). The combination resulted in increased cytotoxicity in MDA-MB-468 and HCC38 where cytostatic responses were observed with either single agent at similar concentrations (Fig. 4A). Despite the increased cytotoxicity and growth inhibition, the effect of the combination was primarily additive on growth inhibition in all breast cancer cell lines tested using either model of synergy (Fig. 4B, C). Likewise, ovarian cancer cell lines demonstrated increased sensitivity to both single agents compared to the 10-day double titration matrix proliferation study (Fig. 4D, Supplemental Fig. 5). Increased cytotoxicity was observed in OV-90 where the same concentrations of GSK3326595 or niraparib elicited a cytostatic response (Fig. 4D). Similar to breast cancer cell lines, the combination was mostly additive in this assay format (Fig. 4E, F). Additive effects observed in both tumor types could be due to the decreased window of growth by either single agent at lower concentrations relative to the 10-day double titration proliferation assay. Overall, the increased exposure time and environmental stress increased the sensitivity of breast and ovarian cancer cell lines to inhibition of PRMT5, PARP, and the combination.

### Increased growth inhibitory effects of the combination of PRMT5 and PARP inhibition in breast and ovarian cancer patient derived models

**Fig. 5:**
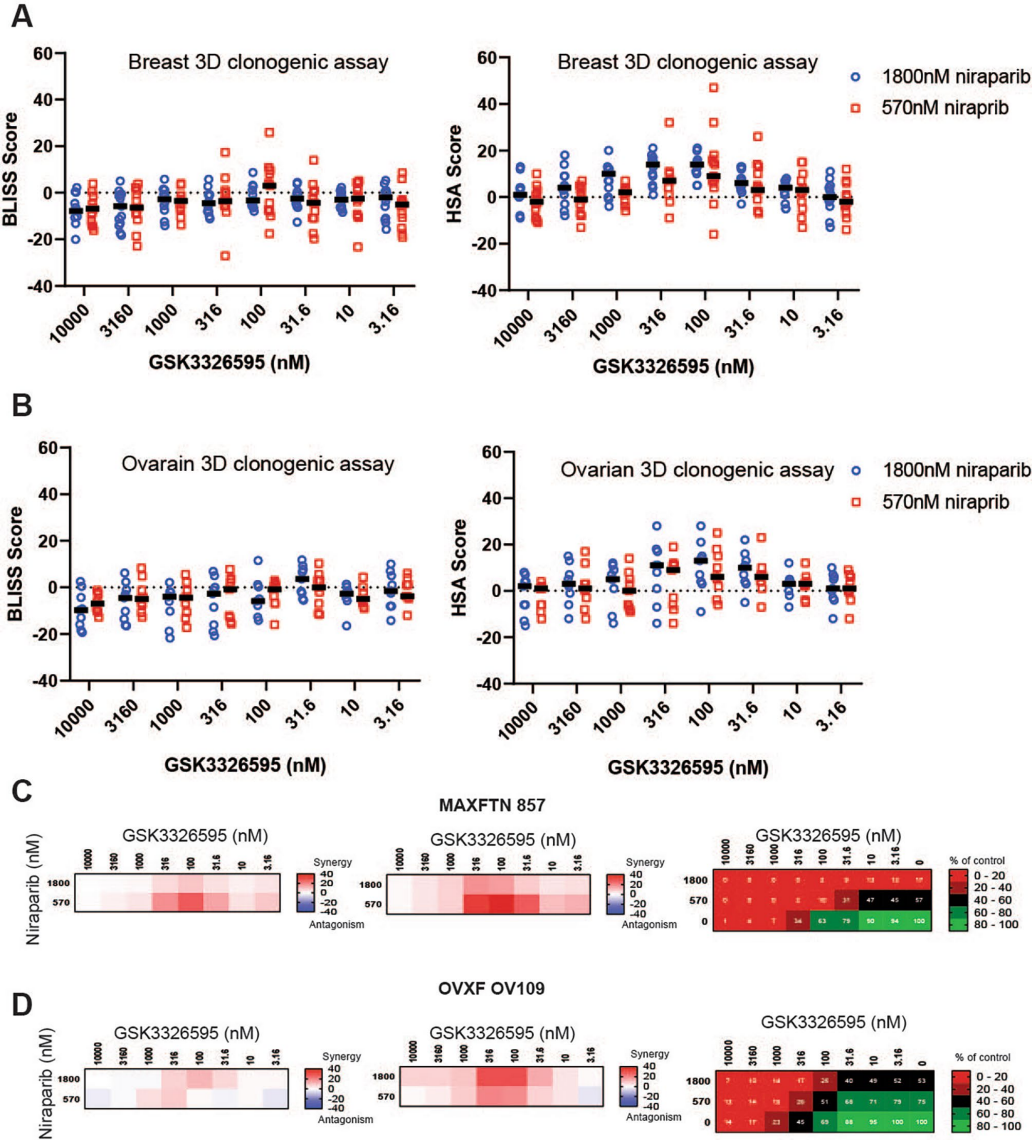
Enhanced growth inhibition by the combination of inhibitors of PRMT5 and PARP in a subset of 3D clonogenic PDX breast and ovarian cancer models. **A**, Average excess over Bliss and HSA score of breast 3D clonogenic in vitro PDX models of breast cancer treated with a titration of GSK3326595 in combination with either 570 nM (red squares) or 1800 nM (blue circles) niraparib. Black bars represent the median score at each concentration of GSK3326595 in combination with niraparib. **B**, Average excess over Bliss and HSA scores of in vitro PDX models of ovarian cancer treated with a titration of GSK3326595 in combination with either 570 nM (red squares) or 1800 nM (blue circles) niraparib. Black bars represent the median score at each concentration of GSK3326595 in combination with niraparib. **C,D**, Average score of excess over bliss and HSA on growth inhibition and average percent of growth relative to control in the MAXFHER 857 breast cancer model (C) and OVXF OV109 ovarian cancer model (D)

Patient derived xenograft (PDX) models have been utilized as a powerful tool to better study the characteristics of tumors and predict drug sensitivity. Whereas the extended culture of cancer cell lines may lead to secondary molecular changes, PDX models better retain the orginal characteristics of the patient tumors [ref. 46]. Therefore, we tested the combination of niraparib with GSK3326595 in 13 breast and 9 ovarian cancer PDX models using an in vitro 3D spheroid clonogenic assay. This method measures anchorage independent growth of spheriods from a single cell suspension of PDX tumors in semi-solid media, and has been found to be highly predictive of the efficacy of targeted agents in in vivo models [ref. 47]. Established 3D spheroids were treated with a titration of GSK3326595 at fixed concentrations of niraparib for 8 to 13 days, depending on the model, and colony viability was evaluated. Treatment with GSK3326595 alone decreased viable colony number across multiple models, where greater than 50% maximal inhibition with an IC_50_ less than 1 µM was observed in more than half of the models tested (Supplemental Fig. 6A). Treatment with niraparib alone demonstrated more limited effects on the PDX models, where greater than 50% inhibition was observed in 6 out of 22 PDX models at the highest concentration of niraparib tested (Supplemental Fig. 6B). Sensitivity to either GSK3326595 or niraparib did not correlate with *BRCA1* or *BRCA2* mutation status or tumor type (Supplemental Fig. 6). In both breast and ovarian cancer PDX models, synergy was only observed using the least stringent HSA model (Fig. 5A-B, Supplemental Figs. 7, 8). Among models with synergy by HSA, the combination showed the strongest benefit in inhibiting proliferation of the MAXFHER 857 and OVXF OV109 models (Fig. 5C-D, Supplemental Figs. 7, 8). Together, these data demonstrate that the combination of PRMT5 and PARP inhibitors increase anti-proliferative activity, with effects ranging from additive to synergistic by HSA, depending on the model and assay format utilized.

### In vivo efficacy of PRMT5i and niraparib combination in breast and ovarian xenograft tumor models

**Fig. 6:**
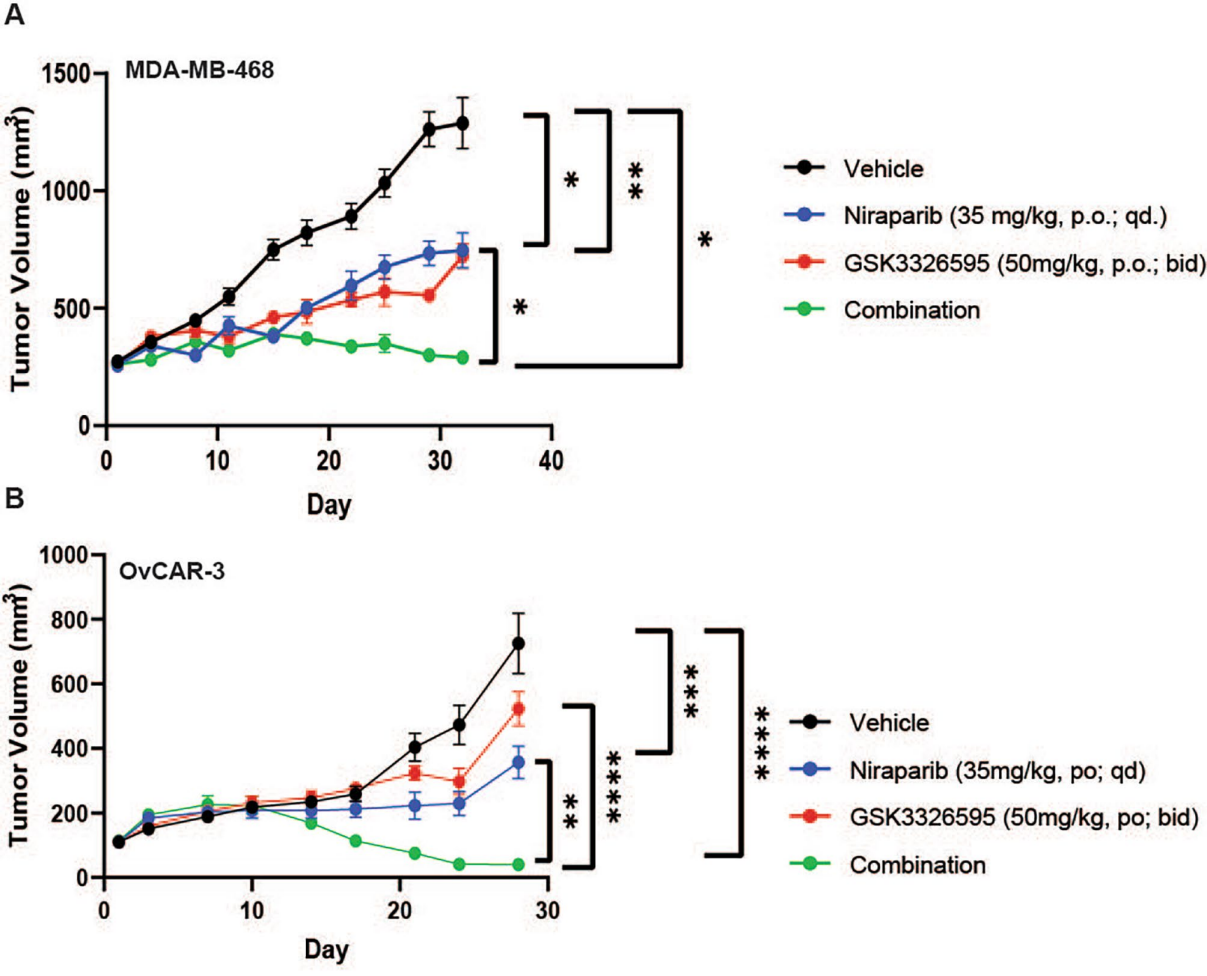
Inhibitio*n* of in vivo tumor growth inhibition of breast and ovarian cancer by GSK3326595, niraparib, or the combination. **A**, Efficacy of GSK3326595, niraparib, or the combination in the MDA-MB-468 xenograft model. NSG mice bearing established MDA-MB-468 tumors were treated with either vehicle (0.5% methylcellulose), twice daily oral administration of GSK3326595 (50 mg/kg), once daily oral administration of niraparib (35 mg/kg), or the combination of both agents (n = 10). **B**, Efficacy of GSK3326595, niraparib, or the combination in the OVCAR3 xenograft model. CD.17 SCID mice bearing established OVCAR-3 tumors were treated with either vehicle, twice daily oral administration PRMT5i (50 mg/kg), once daily oral administration of niraparib (35 mg/kg), or the combination of both agents (n = 10). All error bars represent mean ± SEM and student t-test determined significance. * p ≤ 0.05; ** p ≤ 0.01; *** p ≤ 0.001; **** p ≤ 0.0001

To determine how the anti-proliferative activity of the PRMT5i and PARPi combination observed in vitro would translate to tumor growth inhibition in vivo, the efficacy of the combination was evaluated in ovarian and breast cancer cell line xenografts. Mice bearing either MDA-MB-468 (breast cancer) or OVCAR-3 (ovarian cancer) xenografts were dosed with GSK3326595 or niraparib, alone or in combination. As monotherapies, the treatment with niraparib or GSK3326595 resulted in significant tumor growth inhibition in the MDA-MB-468 model (Fig. 6A). However, GSK3326595 dosed in combination with niraparib resulted in the significant inhibition and eventual tumor stasis relative to either single agent or the vehicle treatment group (Fig. 6A).

In the OVCAR3 model, niraparib as a single agent produced a significant reduction in tumor growth relative to the vehicle and GSK3326595, while GSK3326595 had no significant effect on tumor volume (Fig. 6B). However, the combination treatment of GSK3326595 and niraparib resulted in the significant reduction of tumor volume relative to all other dosing groups. Furthermore, 9 out of 10 tumors in animals dosed with the combination were nearly completely eradicated, suggesting a cytotoxic effect to combining PARP and PRMT5 inhibition in vivo (Fig. 6B, Supplemental Fig. 9A). The combination was well tolerated in both xenograft models, body weight loss did not exceed 20% in MDA-MB-468 and body weight loss was not observed in OVCAR3 (Supplemental Fig. 9B). Together, these data demonstrate that the combination of PRMT5i and PARPi can produce nearly complete tumor stasis or regression in models where equivalent doses of each have modest effects.

## Discussion

Deficiency in the HR pathway is hypothesized to be the key mechanism for conveying sensitivity to PARP inhibitors [ref. 48]. Therefore, therapies that modulate the HR pathway in tumors may provide one viable approach for sensitizing cancer cells to PARP inhibition. In this report, we combine a clinically approved PARP inhibitor with a potent, selective inhibitor of PRMT5 to demonstrate increased anti-tumor activity in vitro and in vivo. Previous reports have identified specific PRMT5 substrates that regulate DNA repair, as well as combinations with PRMT5 inhibitors with DNA damaging agents [refs. 27, 49–54]. We have expanded on these findings by evaluating activity of clinically utilized agents in a broader panel of cell lines, demonstrating a combination of mechanisms can improve efficacy. Notably, these cell lines were all proficient for the homologous recombination pathway, suggesting PRMT5 inhibition can broaden the efficacy of PARP inhibitors outside of currently approved indications.

PRMT5 inhibition did not cause a reproducible shift from HR to NHEJ markers in the cell lines tested, suggesting that the choice between NHEJ and HR repair pathways was not consistently affected. Since 53BP1 and RAD51 are early markers of NHEJ and HR, respectively, our data does not rule out the possibility that later steps of each repair process have been compromised by PRMT5i [ref. 55]. Several mechanisms may explain the observed increase of DNA damage upon PRMT5 inhibition, including the induction of DNA damage or the failure to repair DNA breaks that accumulate during DNA replication or transcription. For example, inhibition of PRMT5 leads to broad effects on splicing and methylation of the DDX5 RNA helicase, which could lead to the stabilization of DNA:RNA hybrids (R-loops) [refs. 21, 22, 56, 57]. R-loops form during transcription as the nascent RNA hybridizes with the DNA template to form a three stranded structure. When unresolved, R-loops can result in the accumulation of DNA damage [refs. 58, 59]. In addition, the resolution of DNA damage and subsequent activation of DNA damage checkpoints may be altered by PRMT5 inhibition. PRMT5 mediated methylation and subsequent stabilization of 53BP1 promotes cell survival after DNA damage [ref. 26]. Destabilization or inhibition of 53BP1 inhibits NHEJ and results in apoptosis after DNA damage [ref. 60]. Beyond 53BP1, PRMT5 regulates DNA damage repair and resolution through the methylation of transcription factors, histones, and E3 ligases [refs. 26, 49, 50, 51]. However, the precise mechanisms of PRMT5i-induced DNA damage are challenging to elucidate due to the numerous alternative splicing events and methylation substrates regulating DNA damage and repair. Moreover, the variability in DNA damage observed between cell lines tested suggests an underlying complexity that may be mediated by expression levels of PRMT5 substrates among cell lines or variations in the DNA damage sensing and repair machinery preferentially utilized by each cell line. Therefore, identifying the most relevant substrates may lead to the application of appropriate pharmacodynamic biomarkers to follow in patients to predict optimal response to the combination.

In addition to modulating the response to DNA damage, both inhibitors of PARP and PRMT5 have been demonstrated to activate innate immune signaling pathways in cancer cells, inducing expression of immune stimulated genes (ISGs) [refs. 61, 62–65]. In human tumors, increased expression of ISGs is associated with increased immune infiltrate and response to immune checkpoint inhibitors [refs. 66, 67–69]. Therefore, combining these agents may have a synergistic effect on ISG induction, and further potentiate anti-tumor immunity. Assessment of efficacy in an immunocompetent model may reveal an additional mechanism of anti-tumor activity.

The discovery of biomarkers to stratify responding and non-responding patients, along with identification of combination strategies can further expand the benefit of PARP inhibitors to additional patients [refs. 10, 11, 12, 13]. The effect of combining PARP inhibitors with modulation of PRMT5 or Type I PRMTs, responsible for the production of ADMA, has been observed in AML and NSCLC models, respectively [refs. 70, 71]. However, these studies demonstrated combination efficacy in tumor types currently not approved for PARP therapies. Given that the PARP inhibitors niraparib and rucaparib are currently approved for the maintenance of HR proficient cancers, PRMT5 inhibition may represent a combination to expand the utility of PARP inhibition to earlier lines of therapy. Future mechanistic investigation can maximize clinical activity by optimizing dosing regimens to minimize treatment associated toxicities and identifying predictive biomarkers of response to the combination.

### Electronic supplementary material

Below is the link to the electronic supplementary material.

Supplementary Material 1

Supplementary Material 2

Supplementary Material 3

Supplementary Material 4

Supplementary Material 5

Supplementary Material 6

Supplementary Material 7

Supplementary Material 8

Supplementary Material 9

Supplementary Material 10

## Data Availability

All data generated or analyzed during this study are included in this published article [and its supplementary information files].

## Abbreviations

PARP: Poly ADP-Ribose Polymerases
PRMT5: Protein Arginine Methyltransferase 5
NHEJ: Non-Homologous End Joining
HR: Homologous recombination
MMA: Mono-methylated arginine
ADMA: Asymmetric dimethylated arginine
SDMA: Symmetric dimethylated arginine
PDX: Patient Derived Xenograft
GDI: Growth Death Index
BLISS: Bliss independence model
HSA: Highest over single agent
CFA: Colony Formation Assay
CTG: Cell Titer Glo
ISG: Interferon Stimulated Gene
