# Supplementary figures and images for: Inhibiting PRMT5 induces DNA damage and increases anti-proliferative activity of Niraparib, a PARP inhibitor, in models of breast and ovarian cancer
Source: BMC Cancer. 2023 Aug 18;23:775. doi: 10.1186/s12885-023-11260-z (PMC10436459; doi:10.1186/s12885-023-11260-z)

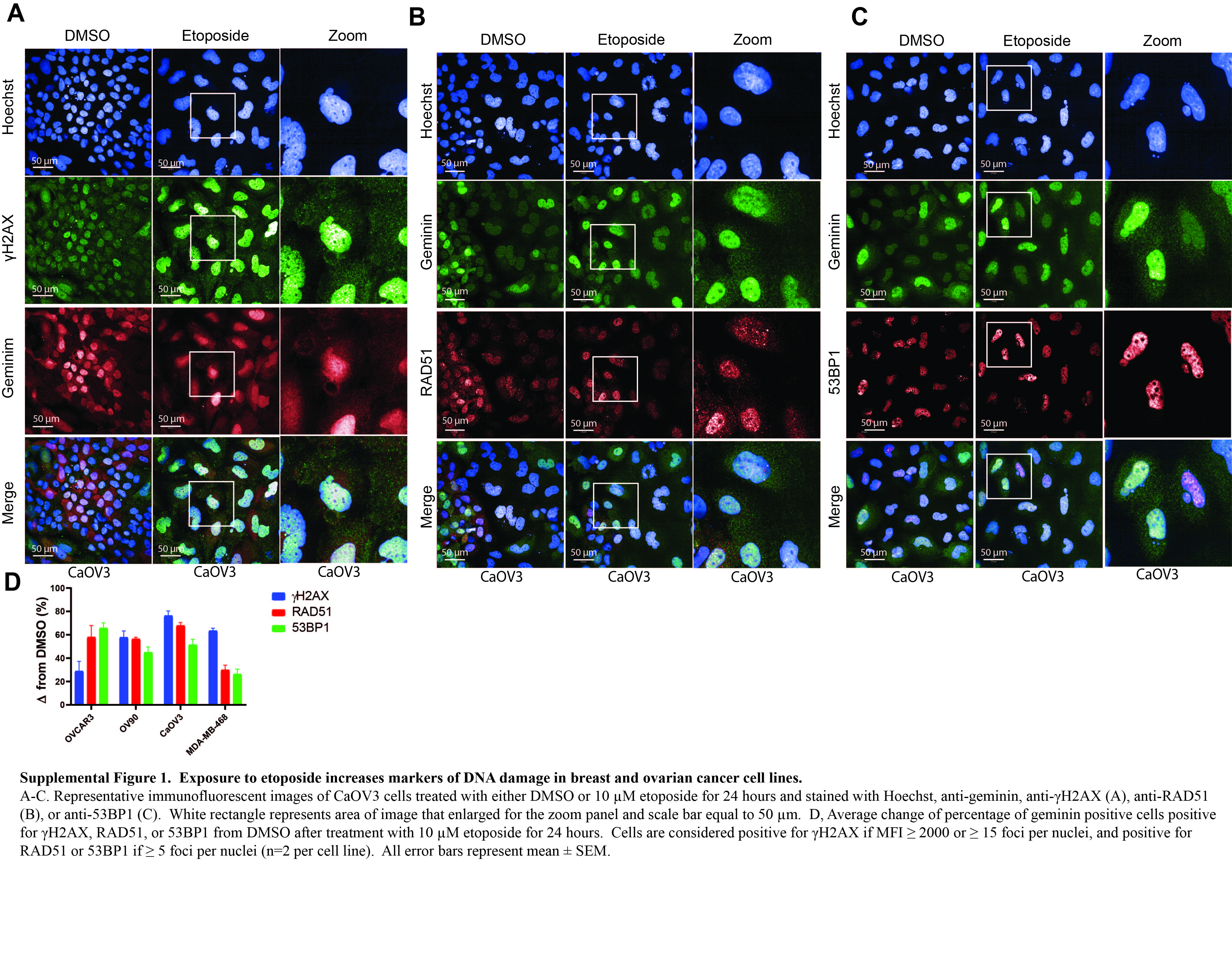

Supplement: Supplementary file 1 — Supplementary Material 1 [file 12885_2023_11260_MOESM1_ESM.jpg]

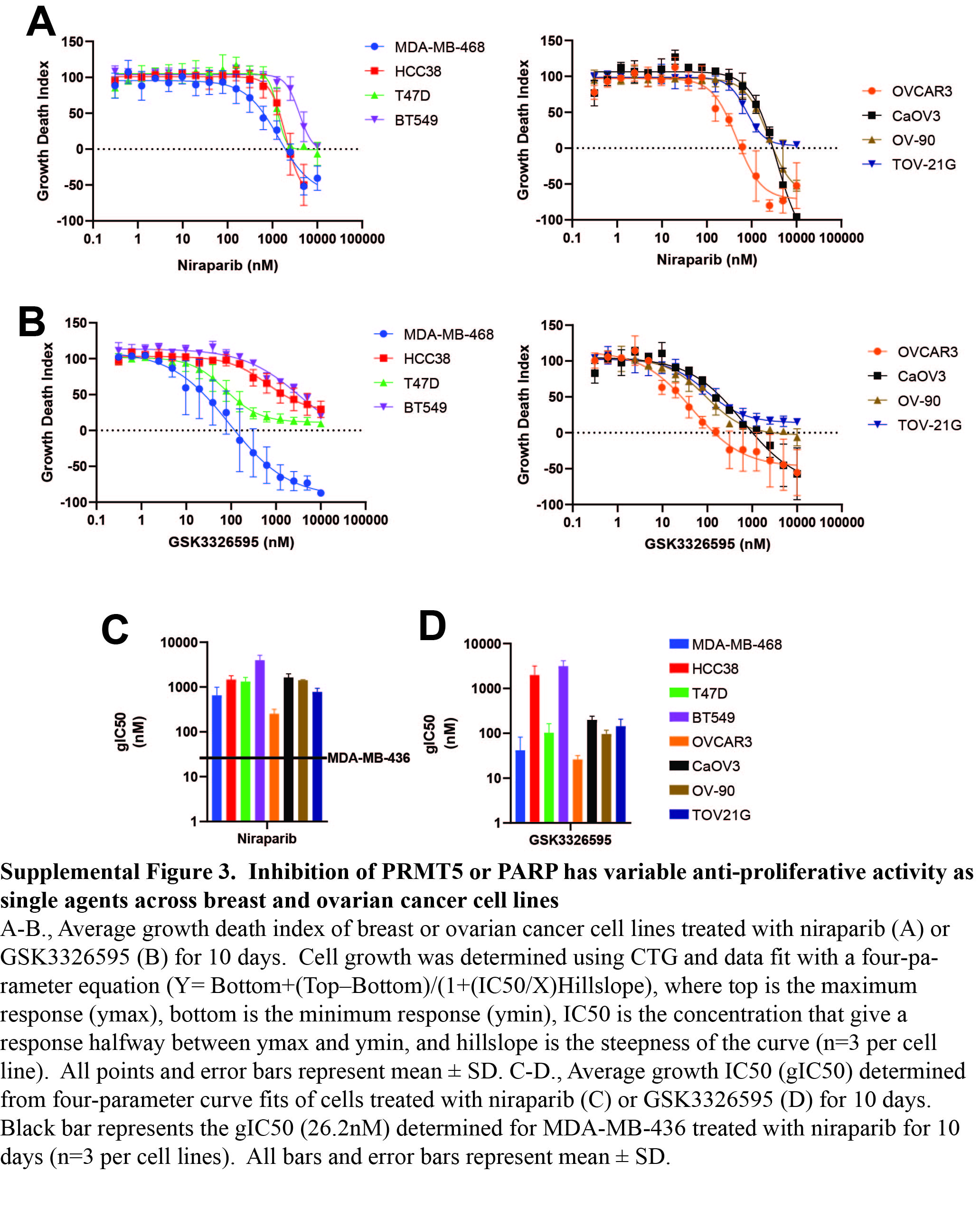

Supplement: Supplementary file 2 — Supplementary Material 2 [file 12885_2023_11260_MOESM2_ESM.jpg]

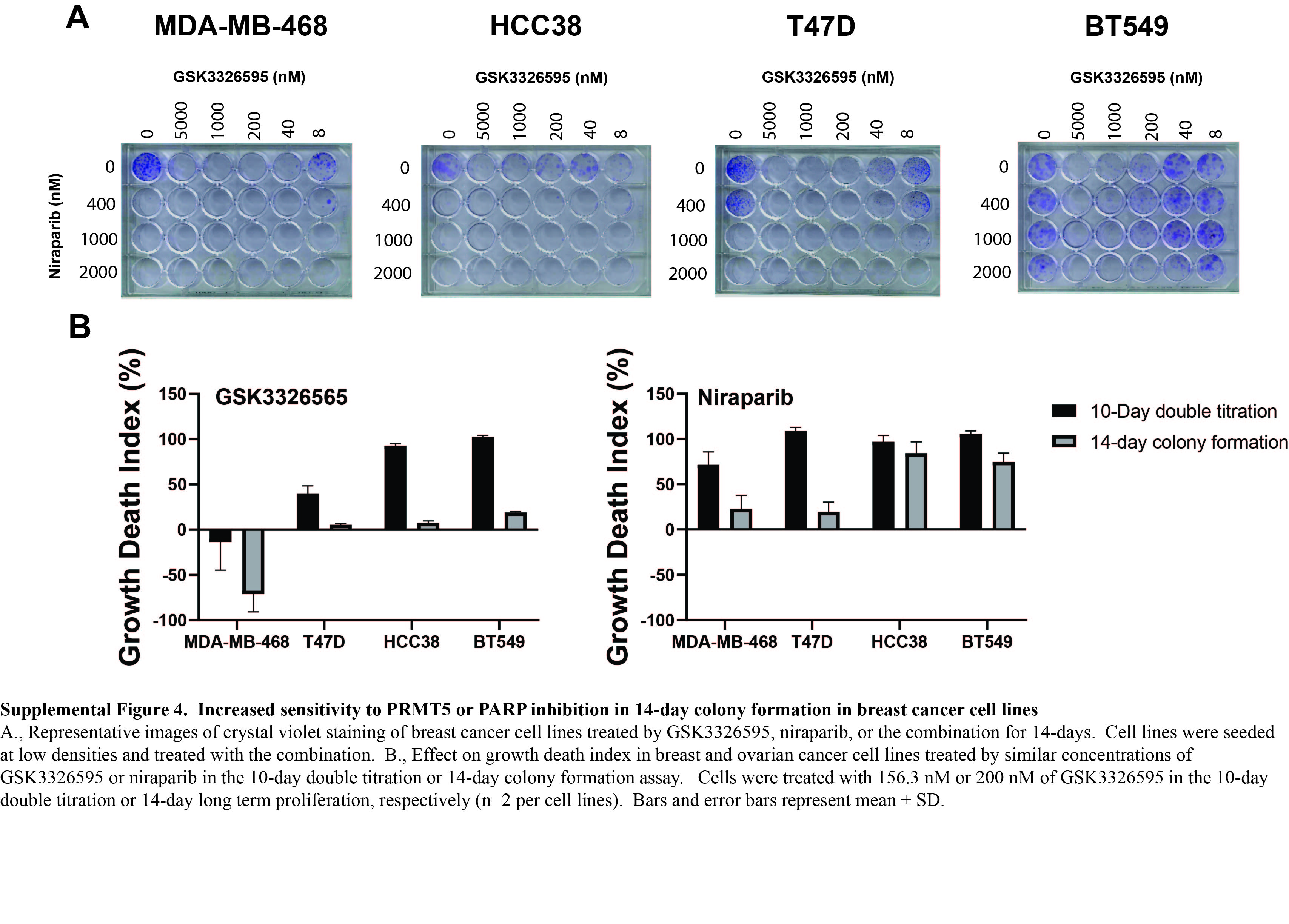

Supplement: Supplementary file 3 — Supplementary Material 3 [file 12885_2023_11260_MOESM3_ESM.jpg]

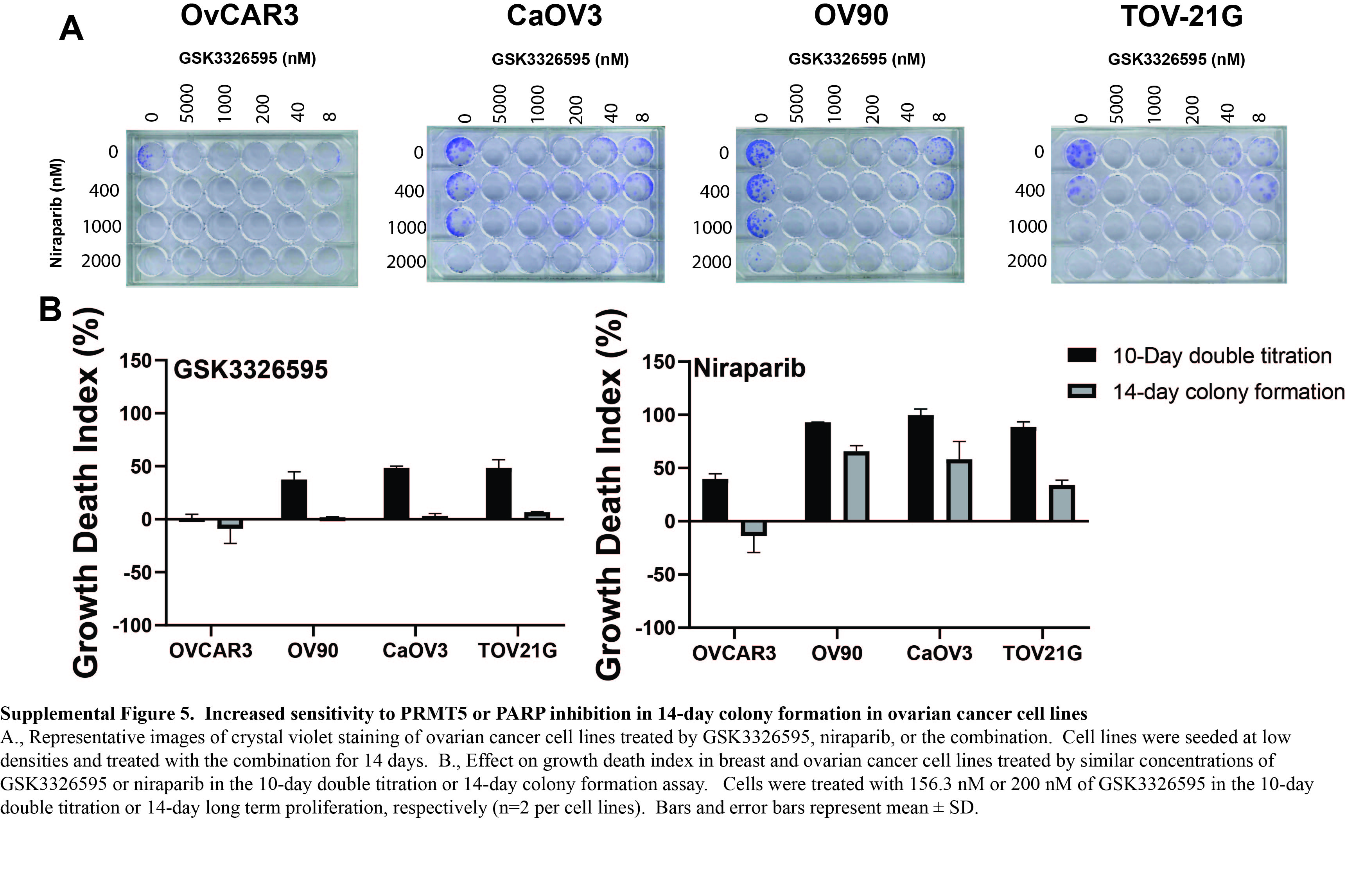

Supplement: Supplementary file 4 — Supplementary Material 4 [file 12885_2023_11260_MOESM4_ESM.jpg]

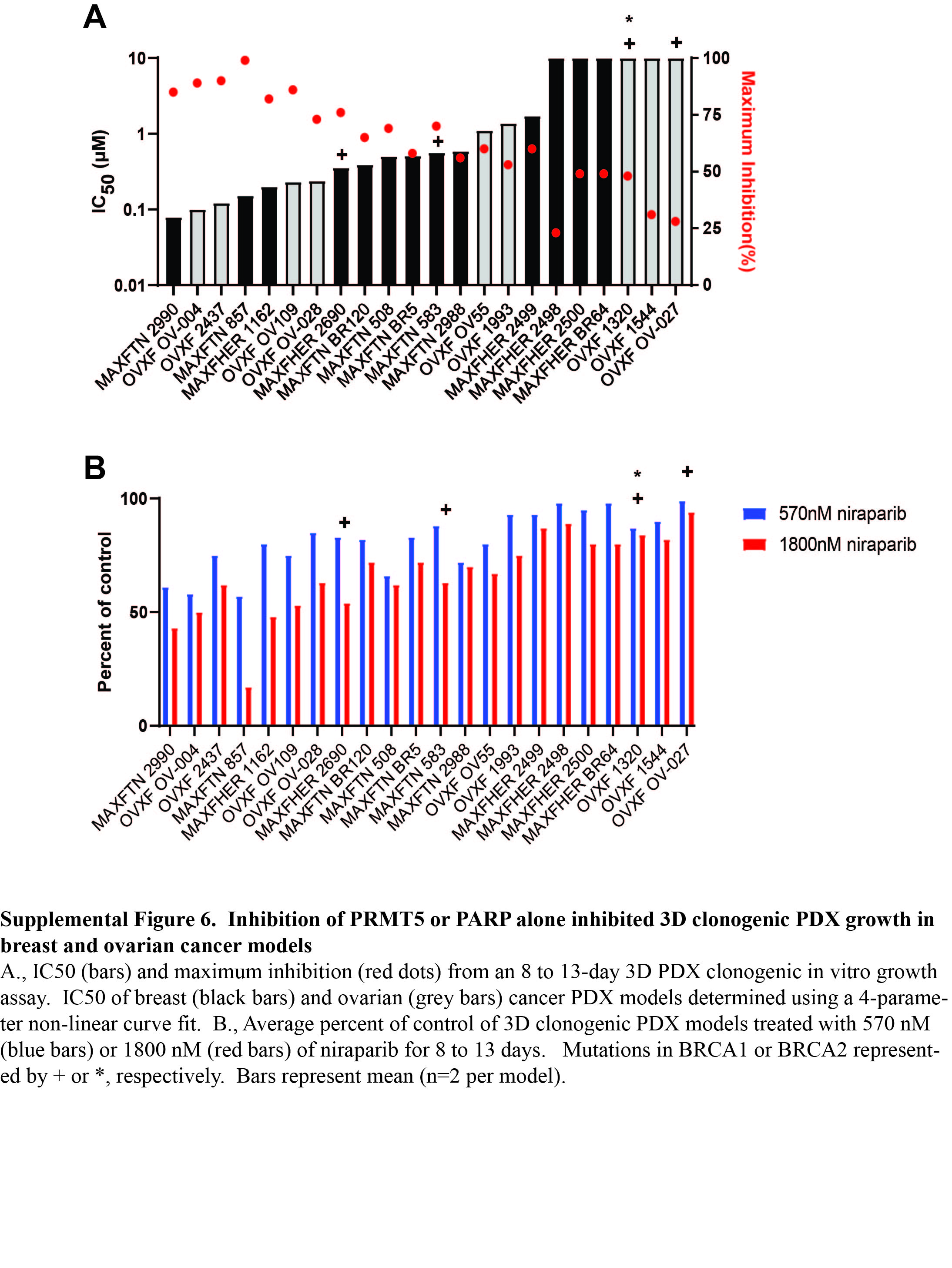

Supplement: Supplementary file 5 — Supplementary Material 5 [file 12885_2023_11260_MOESM5_ESM.jpg]

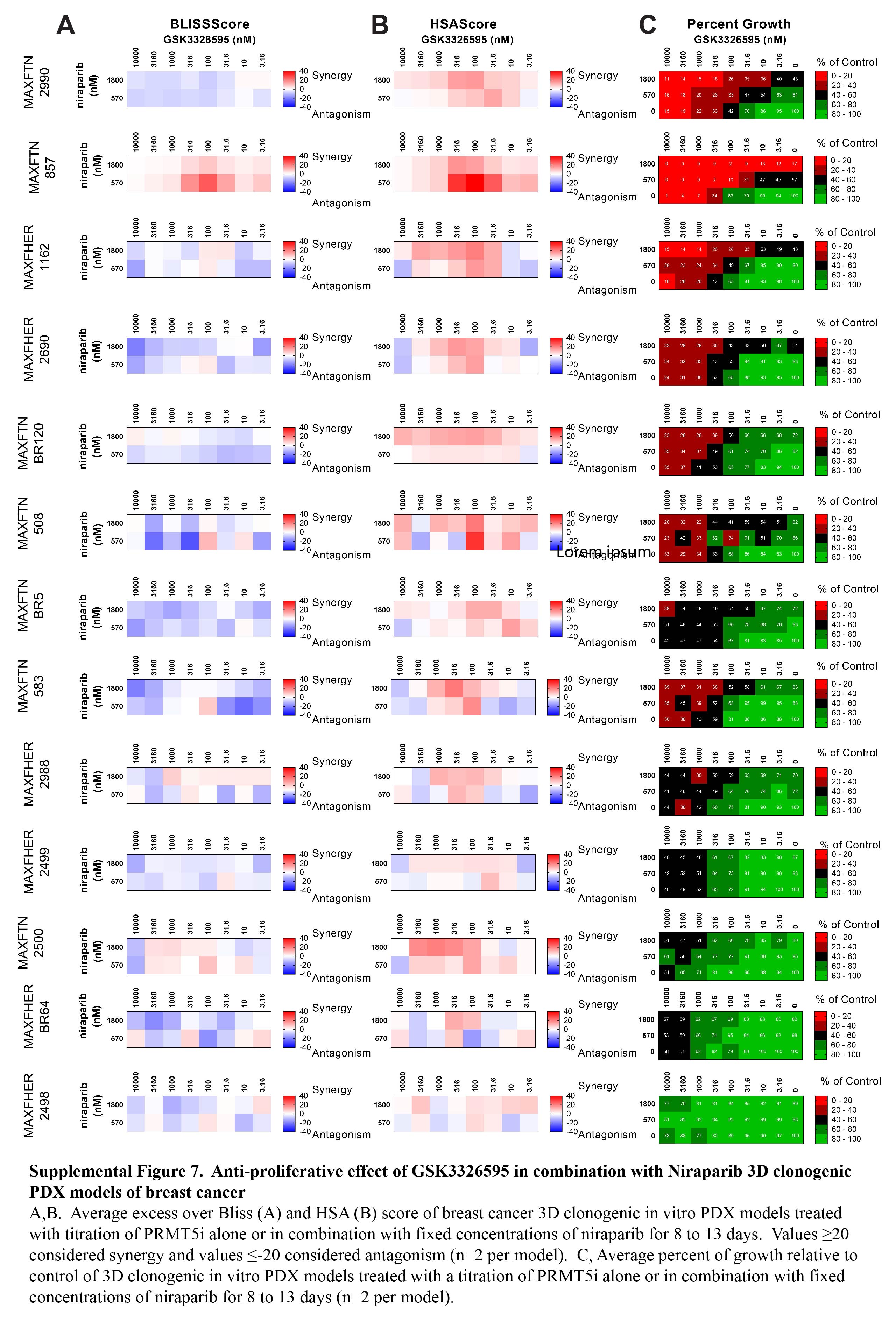

Supplement: Supplementary file 6 — Supplementary Material 6 [file 12885_2023_11260_MOESM6_ESM.jpg]

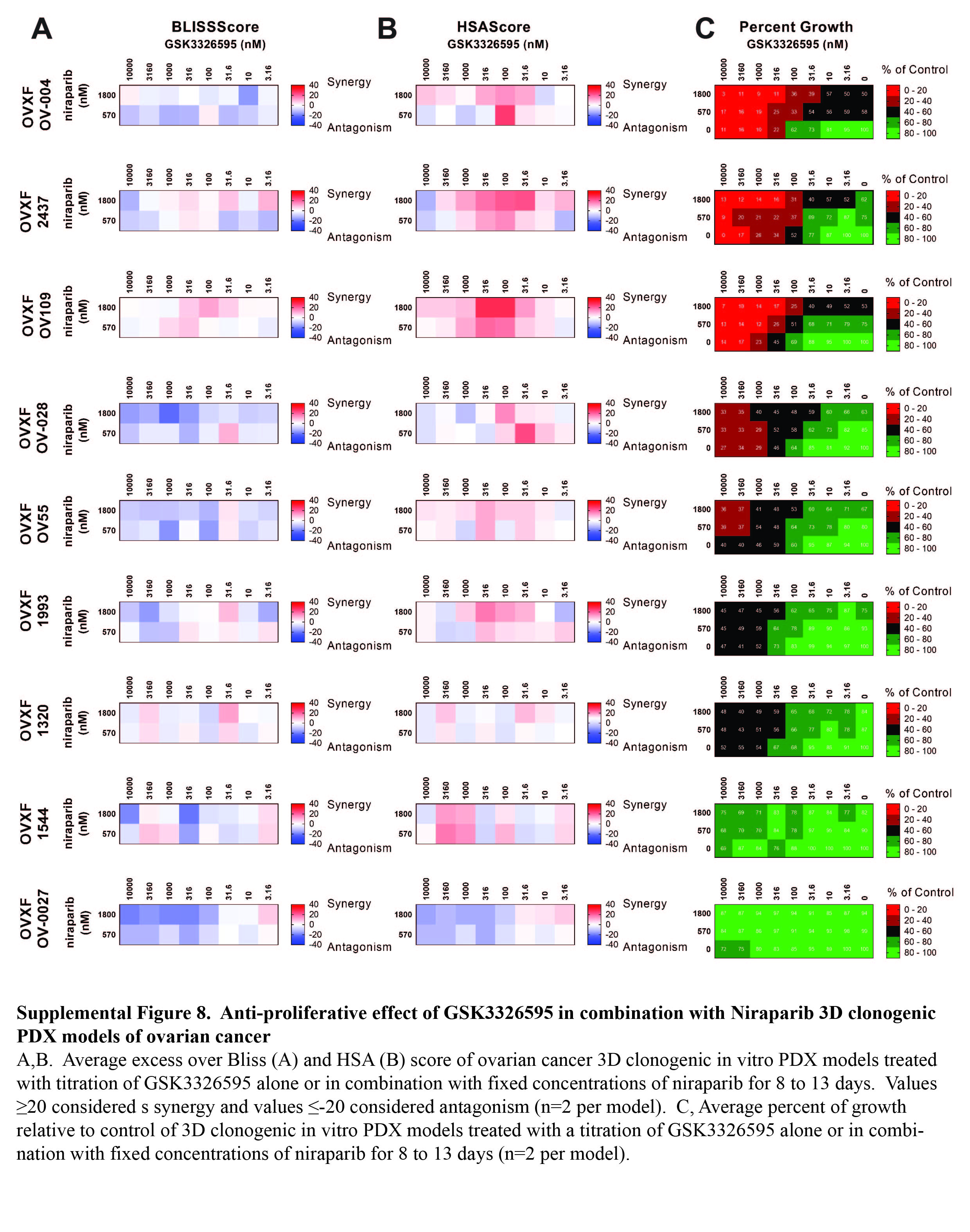

Supplement: Supplementary file 7 — Supplementary Material 7 [file 12885_2023_11260_MOESM7_ESM.jpg]

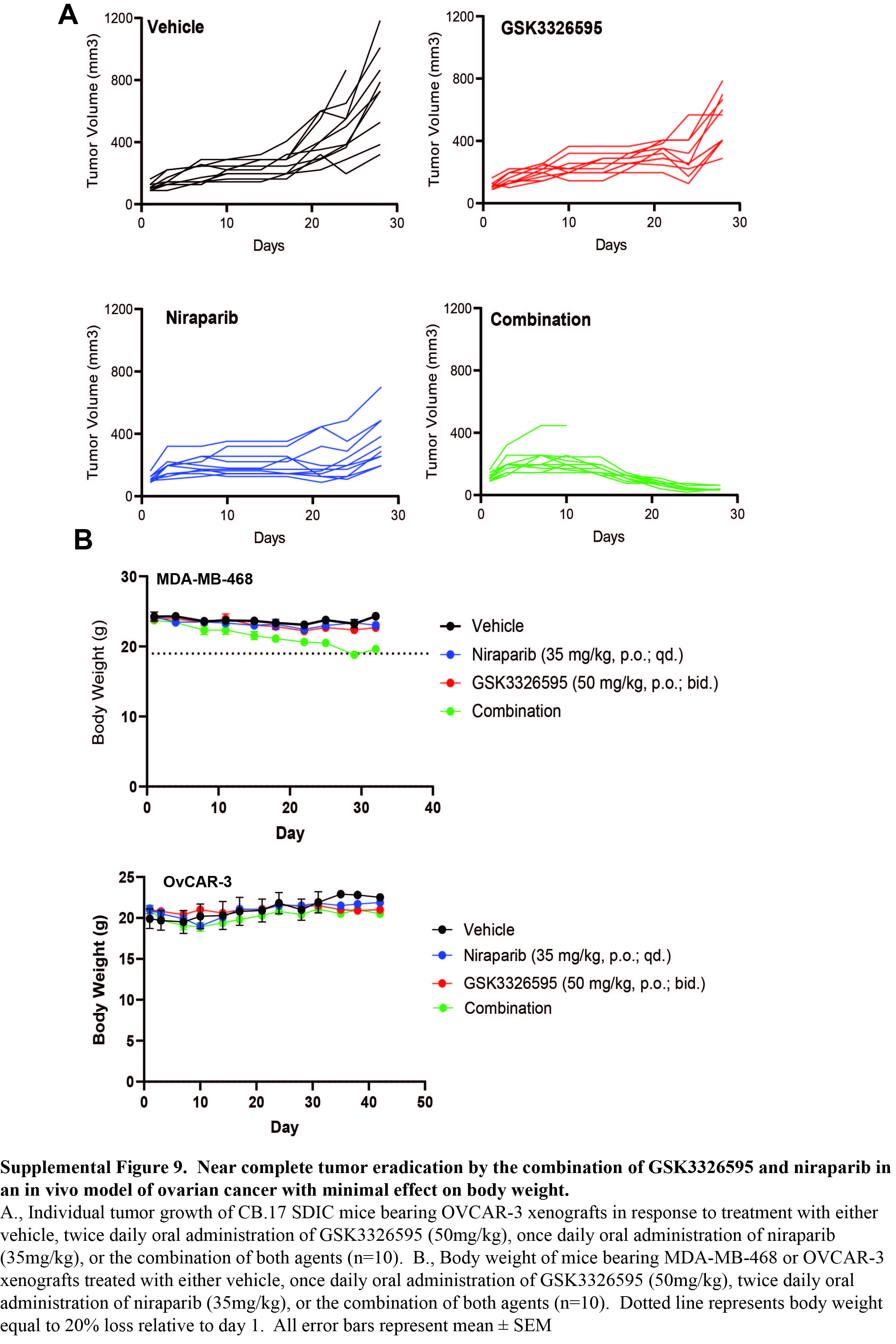

Supplement: Supplementary file 8 — Supplementary Material 8 [file 12885_2023_11260_MOESM8_ESM.jpg]

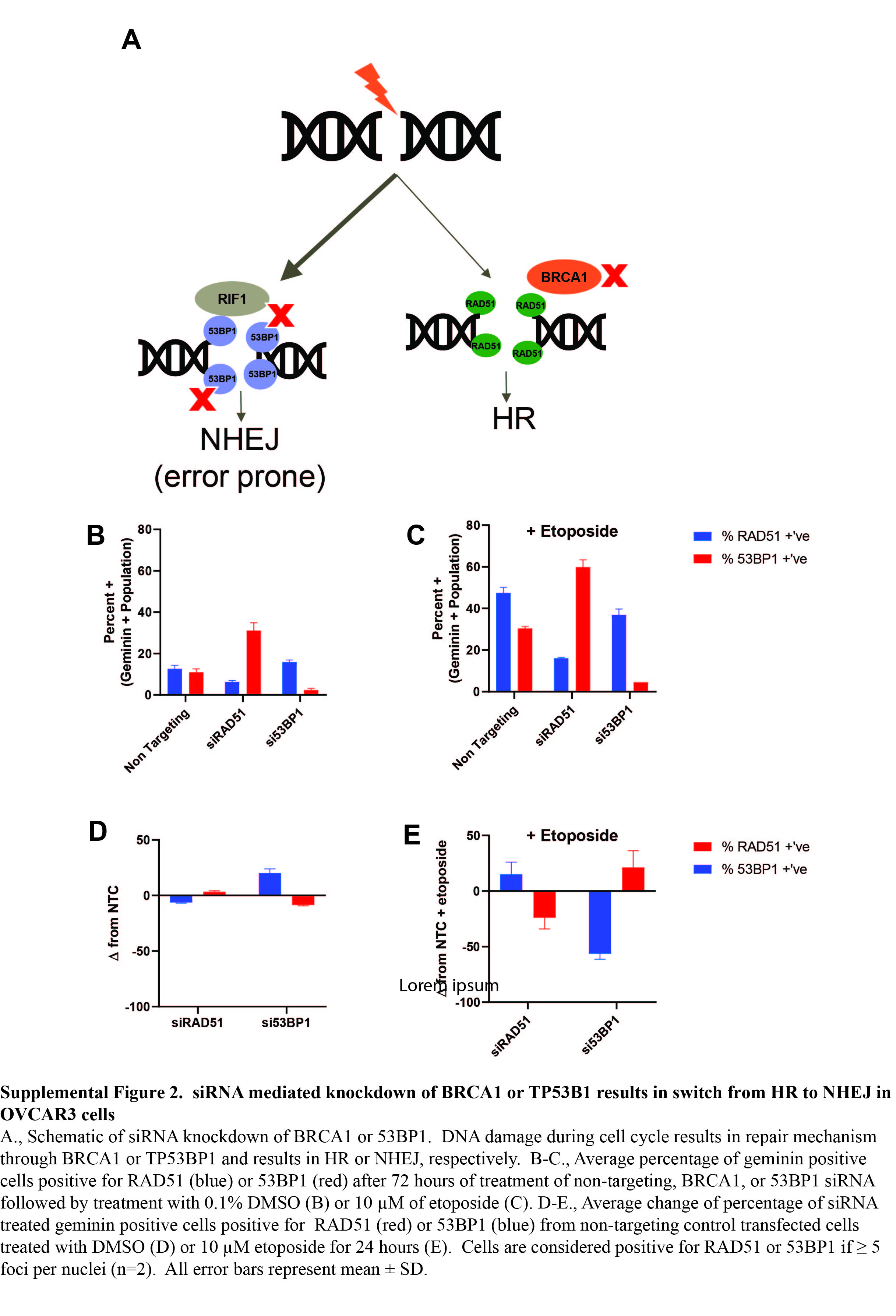

Supplement: Supplementary file 9 — Supplementary Material 9 [file 12885_2023_11260_MOESM9_ESM.jpg]
