# Supplementary material for: Inhibiting PRMT5 induces DNA damage and increases anti-proliferative activity of Niraparib, a PARP inhibitor, in models of breast and ovarian cancer
Source: BMC Cancer. 2023 Aug 18;23:775. doi: 10.1186/s12885-023-11260-z (PMC10436459; doi:10.1186/s12885-023-11260-z)
Supplement: Supplementary file 10 — Supplementary Material 10 [file 12885_2023_11260_MOESM10_ESM.docx]

**Supplementary Table 1. Index of cell lines used throughout manuscript assays**

| **Cell Line** | Immunofluorescence Assay | siRNA-mediated knockdowns | 10-day double titration assay | 14-day long-term proliferation assay | *In vivo* xenograft |
| --- | --- | --- | --- | --- | --- |
| OVCAR3 | ✓ | ✓ | ✓ | ✓ | ✓ |
| CaOV3 | ✓ | 🗶 | ✓ | ✓ | 🗶 |
| OV-90 | ✓ | 🗶 | ✓ | ✓ | 🗶 |
| TOV-21G | 🗶 | 🗶 | ✓ | ✓ | 🗶 |
| MDA-MB-468 | ✓ | 🗶 | ✓ | ✓ | ✓ |
| HCC38 | 🗶 | 🗶 | ✓ | ✓ | 🗶 |
| T47D | 🗶 | 🗶 | ✓ | ✓ | 🗶 |
| BT549 | 🗶 | 🗶 | ✓ | ✓ | 🗶 |

Table of cell lines and assays used throughout manuscript. Checkmark denotes that the cell line was used in the corresponding assay.

**Supplemental Table 2. Expression and mutational status of PRMT5 and DNA damage repair associated gene in breast and ovarian cancer cell lines**

|  | **PRMT5** | | **BRCA1** | | **BRCA2** | | **PARP1** | | **TP53BP1** | | **RAD51** | |
| --- | --- | --- | --- | --- | --- | --- | --- | --- | --- | --- | --- | --- |
| **Cell Line** | **Mutations** | **PRKM** | **Mutations** | **PRKM** | **Mutations** | **PRKM** | **Mutations** | **PRKM** | **Mutations** | **PRKM** | **Mutations** | **PRKM** |
| OVCAR3 | WT | 26.31 | WT | 5.66 | WT | 2.05 | WT | 61.89 | WT | 19.53 | WT | 12.66 |
| CaOV3 | WT | 42.13 | WT | 6.01 | WT | 5.14 | WT | 46.66 | WT | 17.08 | WT | 5.19 |
| OV-90 | WT | 33.05 | WT | 4.67 | WT | 1.11 | WT | 33.06 | WT | 6.25 | WT | 9.71 |
| TOV-21G | WT | 31.52 | WT | 5.64 | WT | 2.07 | WT | 31.36 | WT | 19.74 | WT | 13.24 |
| MDA-MB-468 | WT | 31.63 | WT | 6.79 | M965I | 3.77 | WT | 42.27 | WT | 8.06 | WT | 8.07 |
| HCC38 | WT | 23.06 | WT | 2.50 | K644R | 4.53 | WT | 43.37 | K878E | 19.61 | WT | 11.58 |
| T47D | WT | 32.55 | WT | 12.91 | WT | 1.67 | WT | 63.30 | WT | 19.84 | WT | 13.57 |
| BT549 | WT | 44.79 | WT | 10.16 | WT | 2.51 | WT | 50.50 | WT | 13.82 | WT | 12.11 |

Mutational status and mRNA expression, PRKM, for ovarian and breast cancer cell lines. Mutational status and mRNA expression data analyzed using data previously described (refs. 72, 73, 74).

72. Ghandi M, Huang FW, Jane-Valbuena J, Kryukov GV, Lo CC, McDonald ER, 3rd, et al. Next-generation characterization of the Cancer Cell Line Encyclopedia. Nature. 2019;569(7757):503-8.

73. Gao J, Aksoy BA, Dogrusoz U, Dresdner G, Gross B, Sumer SO, et al. Integrative analysis of complex cancer genomics and clinical profiles using the cBioPortal. Sci Signal. 2013;6(269):pl1.

74. Cerami E, Gao J, Dogrusoz U, Gross BE, Sumer SO, Aksoy BA, et al. The cBio cancer genomics portal: an open platform for exploring multidimensional cancer genomics data. Cancer Discov. 2012;2(5):401-4.
